# Editorial: Emerging topics on chemical safety assessment

**DOI:** 10.3389/ftox.2024.1542469

**Published:** 2025-01-10

**Authors:** Maricel V. Maffini, Laura N. Vandenberg

**Affiliations:** ^1^ Independent Consultant, Frederick, MD, United States; ^2^ School of Public Health and Health Sciences, University of Massachusetts – Amherst, Amherst, MA, United States

**Keywords:** essential use, globally harmonized methods, chemical testing, risk assessment, risk management, regulations, environmental health

Exposure to a myriad of chemicals, starting prior to conception and continuing until death, has likely contributed to an epidemic of non-communicable diseases seen throughout the world. In the US, it is estimated that 129 million people have at least one major chronic disease (Benavidez et al., 2024). Although individual behaviors are acknowledged to play an important role in the development of many of these health conditions, in recent decades, public health researchers have also demonstrated that chemical exposures contribute to both the prevalence and severity of an increasing number of preventable chronic diseases (Muncke et al., 2023). Using animal models, researchers have developed a large body of evidence demonstrating that synthetic chemicals can cause adverse health effects, especially when exposures occur during sensitive windows of development. Epidemiology and observational wildlife studies similarly show associations between exposures to chemicals from a wide variety of sources and harmful health effects (Woodruff et al., 2023).

In the United States, regulatory agencies have approved tens of thousands of chemicals for uses ranging from agricultural and industrial purposes to food and personal care products, and other consumer goods (Wang et al., 2020). For many of these chemicals, exposures to human populations are ubiquitous. Many of them have been allowed to be used with little or no scrutiny from regulatory agencies. Using food as an example, less than 40% of the chemicals the US Food and Drug Administration has allowed to be included in Americans’ diets have enough data to estimate the amount that would be safe for the public to ingest (Neltner et al., 2013); the vast majority of chemicals allowed in food lack reproductive or developmental toxicity data.

The presence of these chemicals in the bodies of people, combined with the adverse effects they cause in controlled animal studies, as well as their association with adverse health outcomes in human populations, suggest that the approaches used to protect public health from hazardous chemicals are insufficient for these purposes (Maffini and Vandenberg, 2017). There are failures in the testing strategies used to evaluate chemicals, the analysis of hazard data by regulatory agencies (Sass et al.), as well as the approaches used to manage risks (Maffini and Vandenberg).

Numerous emerging topics on chemical safety assessment need attention from the scientific community, regulators, and the regulated industries. These include:

Better use of available hazard assessment data: The mammary gland is sensitive to chemical exposures; however, the organ is rarely analyzed properly in regulatory toxicology testing (Kay et al., 2022). A recent study found 76 potential breast carcinogens migrating from food contact materials sold globally (Parkinson et al.). Science-based policy amendments addressing weaknesses in hazard assessment could be an opportunity for breast cancer prevention.
Modernization of hazard assessments: The endpoints commonly measured in conventional toxicology have not kept up with scientific advances. These can include the tools used to measure epigenetic modifications induced by chemical exposures, effects of pollutants on the microbiome, outcomes relevant to endocrine disruption, and complex neurobehavioral outcomes associated with human conditions such as autism spectrum disorder. It should not be assumed that mechanistic data are not also evidence for adversity. For example, developmental exposure to glyphosate and glyphosate-based herbicides contributed to failed embryonic implantation through a mechanism likely to involve genetic and epigenetic modifications in the uterus (Lorenz et al.).
Improving exposure assessments and environmental monitoring: Improving risk assessments and risk management requires more than improved hazard assessment; many updates to exposure assessments are also needed (Vandenberg et al., 2023). In settings where complex chemical mixtures (e.g., pesticides, detergents, disinfection byproducts) are expected, rapid screening techniques could be used to measure discharge of endocrine active compounds and prevent inadvertent contamination of environmental matrixes (Aneck-Hahn et al.).
Phasing out the worst actors: The concept of “essential use” is a decades-old approach to minimize and phase-out toxic chemicals (Protocol, 1987). More recently, the European Commission announced it would apply the same concept that eliminated the use of ozone-depleting chemicals to prioritize for phase-out “the most harmful” chemicals (European Commission: Directorate-General For EU et al., 2023). An analysis of 100 of ECHA’s REACH authorization applications for information needed to qualify substances’ uses as “essential” identified major challenges in this categorization including a lack of clear detailed use information and guidance for the applicants (Borchert et al.).
Using globally harmonized methods to classify chemicals, regardless of their use: One of the critiques of regulatory agencies is that a chemical can be considered too hazardous for use in one sector but allowed for use in other products (e.g., toys vs. food). The Globally Harmonized System (GHS) of Classification and Labelling of Chemicals is a useful tool to enhance protections to human health and the environment if they can achieve the aim of “one substance, one assessment”. In the European Union, for example, the classification, labeling and packaging of chemicals can be used to improve risk management and chemical regulations (Kättström et al.). However, even these straightforward approaches need to be continuously improved to avoid ambiguity in how hazard labels are interpreted, and whether self-classification by chemical manufacturers uses appropriate evidence and is consistent between companies.

Scientific contributions from many different disciplines and fields have improved our knowledge of the effects chemical exposures have on chronic diseases of increasing concern to human populations. Stalled agencies, under pressure from industry, have halted regulatory progress and allowed exposures to harmful chemicals and pollutants to continue. Conflicts of interest, changes in governmental administrations, and a regulatory ecosystem that is resistant to change are also contributing to the inertia that plagues regulatory toxicology. When regulations fail to protect the public, other remedies can be available, including educational and advocacy campaigns designed to shift consumer behaviors away from the use of hazardous chemicals, citizen petitions to push regulatory agencies to take action, and use of the judicial system to hold both regulatory agencies and polluting industries accountable for their failure to act and the harm they inflict (Maffini and Vandenberg).

Ultimately, the best available scientific evidence must be used to support decision-making by regulatory authorities, and their decisions should be reviewed with the latest and best scientific evidence in mind. Approaches to toxicity testing, exposure assessment, risk assessment, and risk management *should* change with time. A static regulatory system puts human and environmental health at risk.
